# Differential Gene Expression and Protein–Protein Interaction Networks in Bovine Leukemia Virus Infected Cattle: An RNA-Seq Study

**DOI:** 10.3390/pathogens14090887

**Published:** 2025-09-04

**Authors:** Ana S. González-Méndez, Mohammad Mehdi Akbarin, Fernando Cerón-Téllez, Gabriel Eduardo Acevedo-Jiménez, Cecilia Rodríguez-Murillo, Víctor David González-Fernández, Lucero de María Ávila-De la Vega, Marisela Leal-Hernández, Hugo Ramírez Álvarez

**Affiliations:** 1 National Center for Disciplinary Research in Animal Health and Food Safety, National Institute of Forestry, Agriculture and Livestock Research (INIFAP), Mexico City 05110, CP, Mexico; 2 Virology, Genetics, and Molecular Biology Laboratory, Faculty of Higher Studies Cuautitlán, Veterinary Medicine, Campus 4, National Autonomous University of Mexico, Cuautitlan Izacalli 54714, EM, Mexico; 3 Medical School, Mashhad Medical Sciences, Islamic Azad University, Mashhad 9187147578, Iran

**Keywords:** bovine leukemia virus, asymptomatic carrier, persistence lymphoma, RNA-seq, protein–protein interaction networks

## Abstract

Introduction: Bovine leukemia virus is a single-stranded RNA virus that targets B cell CD5^+^ lymphocytes in cattle. Only a tiny percentage of individuals develop malignant lymphoproliferative disorders, while most remain healthy carriers or experience persistent lymphocytosis. The exact mechanisms leading to lymphoma development are complex and not fully understood. RNA-seq analysis of cows’ peripheral blood leukocytes (PBLs) with and without Bovine leukemia virus (BLV) antibodies was conducted to gain a deeper understanding of molecular events beyond BLV infection. Method: Eighteen samples were selected, and their RNA was sequenced. For gene expression analysis and protein–protein network interactions, three groups were selected, including healthy negative samples (CT, n = 7), asymptomatic carriers (AC, n = 5), and persistent lymphocytosis (PL, n = 6), to provide the differentially expressed gene (DEG) and protein–protein interaction network (PPIN) outputs. Results: Our results demonstrated that in comparison to CT, ACs upregulated TLR7 and transcription activation factors. In the CT vs. PL group, *MHC class II*, transcription activation factors, and anti-inflammatory cytokines increased, while the acute-phase proteins, antiviral receptors, and inflammatory cytokines decreased. Additionally, antiviral receptors, acute-phase proteins, and inflammatory receptors were downregulated in the PL versus the AC groups. Moreover, PPINs analysis suggested that nuclear receptor corepressor 1 (NCOR1), serine/arginine repetitive matrix 2 (SRRM2), LUC7 like 3 pre-mRNA splicing factor (LUC7L3), TWIST neighbor (TWISTNB), U6 small nuclear RNA and mRNA degradation associated (LSM4), eukaryotic translation elongation factor 2 (EEF2), ubiquitin C (UBC), CD74, and heterogeneous nuclear ribonucleoprotein A2/B1 (HNRNP A2B1) are possible hub gene candidates in the PL group. Conclusions: Our results suggest that innate and cellular immune responses are more loose in severe BLV infectious conditions, while the PPINs revealed that new protein interactions are necessary for oncogenesis.

## 1. Introduction

Bovine leukemia virus (BLV) infection poses a significant global challenge to the cattle industry, affecting productivity and animal welfare [1,2]. This virus belongs to the genus *Deltaretrovirus* within the *Retroviridae* family; like other retroviruses, it possesses a single-stranded RNA genome, as well as the enzymes reverse transcriptase and integrase [3]. This virus is categorized as an enzootic infectious agent, and the most common target cell is the B cell CD5^+^ cattle lymphocytes. Despite the complete eradication of BLV infection in several European regions, the worldwide distribution of the infection continues, extending from China and Japan to North and South American countries, including the United States, Canada, Brazil, and Argentina [1,4]. Additionally, BLV infection was identified in approximately 7% of the general cattle population across 13 municipalities and regions in Mexico [5].

As the provirus integrates into the host genome, BLV infection typically establishes persistent, lifelong infection characterized by asymptomatic persistent lymphocytosis (APL) in 30–40% of infected individuals [6]. The total white blood cell count in persistence lymphocytosis (PL) patients is two or three times higher than expected, which is due to a higher BLV proviral load [6,7]. However, a small percentage of infected animals (estimated at 3–5%) develop malignant lymphoproliferative disorders, including B-cell lymphosarcoma, after at least five years or more of infection. Although the virus can infect diverse immune cell populations, including CD5^+^ and CD5-B lymphocytes, monocytes, and both T helper and cytotoxic lymphocytes, BLV-related cancers predominantly develop from a specific type of B cell, those expressing CD5 and IgM [8].

The remaining infected subjects (60–70%) remain healthy carriers without exhibiting abnormal blood-related manifestations [6,7]. Despite the condition’s low severity, BLV infection causes significant economic losses in the beef and dairy industries due to reduced milk production in infected cattle, lower productivity rates, and increased mortality in cattle [4,6].

The exact mechanisms leading to lymphoma development are complex and not fully understood. The pX region of the BLV genome, located between the *env* gene and the 3′ long terminal repeat (LTR), encodes regulatory proteins that are crucial for viral persistence and pathogenesis [9,10]. This region includes at least two major open reading frames (ORFs), *tax* and *rex*, which produce the Tax and Rex proteins, respectively. Tax is a transcriptional activator that binds to specific enhancer elements within the BLV LTR, primarily targeting the CRE-like motifs through interaction with host transcription factors, such as cAMP response element-binding protein/activating transcription factor (CREB/ATF), thereby promoting viral gene expression [10,11]. Rex regulates the post-transcriptional processing and nuclear export of unspliced and singly spliced viral RNAs, enabling the translation of structural proteins essential for virion production. Additional auxiliary proteins, such as G4 and R3, encoded within the PX region, contribute to viral infectivity and cell-to-cell transmission by modulating host immune responses and enhancing viral replication efficiency. Mutagenesis studies have demonstrated that the deletion or functional inactivation of PX-encoded proteins significantly impairs BLV replication and leukemogenesis, highlighting the PX region as a critical determinant in the viral life cycle and disease progression [12,13]. However, the Tax protein plays a pivotal role in disrupting cellular regulation and promoting uncontrolled cell growth by manipulating cellular signaling, the cell cycle process, cell migration and adhesion, and the immune response through protein–protein interactions [10,14]. Because multiple variations may be involved in the oncogenic pathways of host–virus interactions [15], the application of high-throughput data analysis via RNA-seq and transcriptomics seems necessary to improve the discovery of molecular pathogenesis. Therefore, this study aims to analyze RNA-seq data from BLV-infected individuals with persistent lymphocytosis and asymptomatic carriers compared to healthy non-infected individuals.

## 2. Materials and Methods

### 2.1. Sample Collection

From 130 cows, blood samples were obtained from 12 healthy Holstein dairy cows in their mid-lactation period (third or fourth lactation) from a single dairy farm in Querétaro, Mexico. These cows were under regular veterinary care and showed no signs of illness. All animals were vaccinated against the bovine respiratory virus complex (BRDC), including bovine respiratory syncytial virus (BRSV), bovine parainfluenza virus 3 (BPIV-3), and bovine herpesvirus-1 (BHV-1). The BLV antibodies were detected in 7 out of 12 samples, and infection was confirmed through polymerase chain reaction (PCR) and quantitative PCR (qPCR). In addition, the six BLV infection positive cases were also included, with signs of persistent lymphocytosis and weight loss, as evidenced by at least three samples. The blood samples were collected using a specific needle and tube type (Vacutainer; Becton, Dickinson and Company, Franklin Lakes, NJ, USA) via a coccygeal venipuncture procedure. Immediately after collection, the samples were stored in a cooled container at a controlled temperature of 4 °C and then transported to the laboratory for further analysis. A Corning cell counter (Corning, NY, USA) was used to facilitate cell counting, and all samples were divided into two groups: normal lymphocyte count and high-persistence lymphocyte count.

### 2.2. Peripheral Blood Mononuclear Cell Separation

As BLV infection includes different types of leukocytes, such as B or T-cell lymphocytes and monocytes, and it also affects granulocyte functions, blood samples were drawn via a coccygeal venipuncture procedure into heparinized tubes, centrifuged at 350× *g* for 15 min to separate plasma and peripheral blood leukocytes (PBLs) instead of peripheral blood mononuclear cells (PBMCs). Then, the PBLs were isolated using a lysis solution [16,17]. Plasma and PBLs were stored at −70 °C until analysis. Lymphocyte counts were performed on all study animals (BLV seronegative and seropositive) and classified according to previously established parameters: normal lymphocyte count and high-persistence lymphocyte count.

### 2.3. RNA Purification

For each sample, approximately 5 × 10^6^ cells were resuspended in 350 μL of RNAlater^®^ (Thermo Fisher Scientific, Waltham, MA, USA; cat. no. AM7020) RLT buffer to facilitate cellular disruption based on the recommended protocol, followed by RNA isolation using the FavorPrep™ Total RNA Isolation Kit II (FAVORGEN, Ping-Tung, Taiwan). The FavorPrep™ Kit II Quick-Start Protocol was strictly followed for RNA extraction. The resulting RNA samples were dissolved in RNase-free water and then assessed for concentration and purity using a Thermo Scientific NanoDrop One C microvolume UV-Vis spectrophotometer (Thermo Fisher Scientific, Waltham, MA, USA). All RNA samples met the required concentration and purification standards. Finally, the RNA was stored at −80 °C in RNase-free water.

### 2.4. Seropositivity of BLV Infection and Proviral Load Evaluation

BLV antibody persistence was evaluated for all included samples using an ELISA assay kit (VMRD, Pullman, WA, USA). If positive, the samples were further tested using a PCR test to quantify the proviral load (PVL). The results indicated that 12 of 18 cows tested positive for BLV antibodies. These positive samples were then subjected to q-PCR testing, which measured the PVL in their groups [18].

### 2.5. Sample Selection, Library Preparation, and Sequencing

All eighteen samples were selected for RNA sequencing based on their results for BLV antibody, PVL screening, and clinical manifestations. This selection comprised thirteen seropositive samples, each representing a different PVL category (low and high) and five seronegative samples. A commercial provider handled library preparation, which involved isolating messenger RNA from the total RNA sample using magnetic beads with poly-T oligo attachments. The mRNA was then broken down into smaller fragments, and complementary DNA (cDNA) was synthesized using random hexamer primers and dNTPs. After ligating the adapters, the cDNA underwent size selection, amplification, and purification. Ultimately, the prepared libraries were sequenced on the Illumina NovaSeq 6000 platform (Illumina Inc., San Diego, CA, USA), yielding approximately 29 million read pairs, each 150 base pairs long, with pair end sequencing.

### 2.6. Genome Mapping and Quality Control

The raw sequencing data were stored in FASTQ format for quality assessment. Any adapter sequences and low-quality reads were removed from the data to ensure accuracy. The remaining reads were then aligned to the Ensembl Bos taurus ARS-UCD1.2 reference genome using the HISAT2 (Johns Hopkins University, Baltimore, MD, USA) (http://daehwankimlab.github.io/hisat2/, accessed on 3 August 2022) alignment program. This allowed for the quantification of gene expression, which was reported in terms of fragments per kilobase of transcript sequence per million base pairs sequenced (FPKM). A correlation analysis was performed for three of the BLV-negative, asymptomatic, and persistent lymphocytosis groups to assess the consistency of gene expression across biological replicates. The results showed strong correlations within each group, with all Pearson correlation coefficients exceeding the recommended threshold of 0.92, indicating high-quality experimental conditions.

### 2.7. Differential Expression Gene (DEG) Analysis

We employed the DESeq2R package (version 1.20.0) (Bioconductor project, open-source, USA) (https://bioconductor.org/packages/release/bioc/html/DESeq2.html, accessed on 5 April 2023) for differential gene expression analysis to identify genes with distinct expression patterns. To guarantee precise analysis, the expression data for each group underwent a rigorous processing pipeline. The R package was employed to identify DEGs, and the data were normalized using the Reads Per Kilobase of transcript per million mapped reads (RPKM) protocol. A more relaxed significance threshold was applied to correct the *p*-values using the Benjamini–Hochberg method, which minimizes false discovery rates, increases the number of DEGs, and facilitates network construction. Genes with an adjusted *p*-value of 0.05 or less and a log2 fold-change of at least 1 or −1 were deemed differentially expressed. These DEGs were then pooled for cluster analysis, where hierarchical clustering was applied based on their FPKM values. The minimum expression threshold of FPKM > 1 in at least one sample or transcript per million (TPM) > 0.5 across 50% of samples. To enable comparison across samples, we standardized the gene expression values by converting them to relative Z-scores.

### 2.8. Network Building

We utilized the STRING database (version 11.0) (Swiss Institute of Bioinformatics, Lausanne, Switzerland) (https://string-db.org/, accessed on 20 June 2023) to identify interactions between proteins and considered the RNA gene expression as a possible candidate for protein expression. This database aggregates data from seven sources: physical interactions, functional associations, high-throughput experimental data, genomic context, co-expression, database information, and text-mined data. We then analyzed the resulting protein–protein interaction networks using Network Analyzer in Cytoscape 3.6.1 (National Institute of General Medical Sciences, Bethesda, MD, USA) (https://cytoscape.org/, accessed on 12 May 2023), focusing on the degree of each node, which represents the number of edges connected to it. The genes with higher degrees were identified as hub genes, playing a crucial role in the network.

### 2.9. Deciphering Biological Networks: Uncovering Functional Clusters and Elucidating Cellular Processes

Deciphering the intricate web of molecular relationships is crucial in systems biology. A key strategy to achieve this is to identify functional modules and analyze biological pathways.

Functional modules are molecular teams working in tandem to execute specific biological tasks. Pathways analysis, on the other hand, delves into the sequential interactions and reactions within these modules. This involves reconstructing complex molecular networks that govern biological processes, such as metabolism, signaling, and gene regulation. By examining these pathways, we can identify key regulatory nodes, understand how molecular disruptions impact biological outcomes, and develop novel therapeutic strategies.

The protein–protein interaction network (PPIN) analyzer is a bioinformatics tool that enables scientists to fully understand the relationships between proteins within a biological system. In cancer research, PPINs have played a crucial role in discovering the leading actors involved in carcinogenesis and the evolution of different kinds of cancer.

To this end, a comparative analysis of asymptomatic carriers (ACs) and persistent lymphocytosis (PL) versus a BLV-negative healthy group (CT) cell network was conducted using Gephi (version 0.10.01)’s fast-unfolding clustering algorithm (Gephi Consortium, Paris, France) (https://cytoscape.org/, accessed on 4 November 2022). A sophisticated graph visualization approach, the Fruchterman–Reingold algorithm, was employed to optimize the layout of vertices and edges. This algorithm treats edges as dynamic springs whose stiffness is adjusted to attract or repel vertices, ultimately reaching an equilibrium state that minimizes the system’s total energy. This process effectively structures the graph’s topology, revealing hidden patterns and relationships.

Biologically relevant modules were then selected from the resulting clusters and visualized using Cytoscape (version 3.6.1) to facilitate interpretation. The entire list of expressed genes for each group served as the background for the EnrichR-ChEA transcription factor targets 2022 analysis (https://maayanlab.cloud/Enrichr/, accessed on 25 August 2023), which identified the target genes of transcription factor binding site profiles.

## 3. Results

### 3.1. PVL Results of AC and PL Groups

Based on the PVL examinations between samples, the mean of the proviral load in the asymptomatic carrier was 5.09 × 10^5^ ± 2.04 × 10^5^ copy/µL, and among the persistent lymphocytosis it was 2.34 × 10^6^ ± 1.02 × 10^6^ copy/µL (CI: 95%). From these values, the two groups of high and low PVL were defined.

### 3.2. Differential Gene Expression

The study revealed significant disparities in the genetic blueprints of blood cells infected with BLV compared to those not infected, as well as between lymphocytosis and asymptomatic carriers. In total, 22,797 reads were detected, with 12,415 reads showing increased activity and 10,382 exhibiting decreased activity in AC versus CT ones. To visualize differential gene expression across the stages of BLV infection, volcano plots were generated for three pairwise comparisons: healthy control vs. asymptomatic carriers, healthy control vs. PL, and persistent lymphocytosis vs. asymptomatic carriers (Appendix A, Appendix B and Appendix C). Each plot displays the log2 fold-change (x-axis) against the −log10-adjusted *p*-value (y-axis), allowing for simultaneous assessment of both statistical significance and the magnitude of expression changes. Genes positioned in the upper right and upper left quadrants represent significantly upregulated and downregulated transcripts, respectively (Appendix A, Appendix B and Appendix C).

In the *healthy control* vs. *asymptomatic* comparison, a limited number of differentially expressed genes were detected, suggesting early or subtle transcriptional alterations during asymptomatic infection. In contrast, the healthy control vs. PL group revealed a larger set of differentially expressed transcripts, highlighting pronounced dysregulation associated with disease progression and lymphocyte expansion. The *PL* vs. *asymptomatic* comparison identified genes that may underlie the transition from asymptomatic carriage to persistent lymphocytosis, reflecting molecular mechanisms linked to viral persistence, immune modulation, and early leukemogenic events (Appendix A, Appendix B and Appendix C).

Overall, the volcano plots provide a visual summary of transcriptional shifts across different clinical outcomes of BLV infection, underscoring key genes and pathways that may contribute to host–virus interactions and pathogenesis (Appendix A, Appendix B and Appendix C).

**Figure 1:**
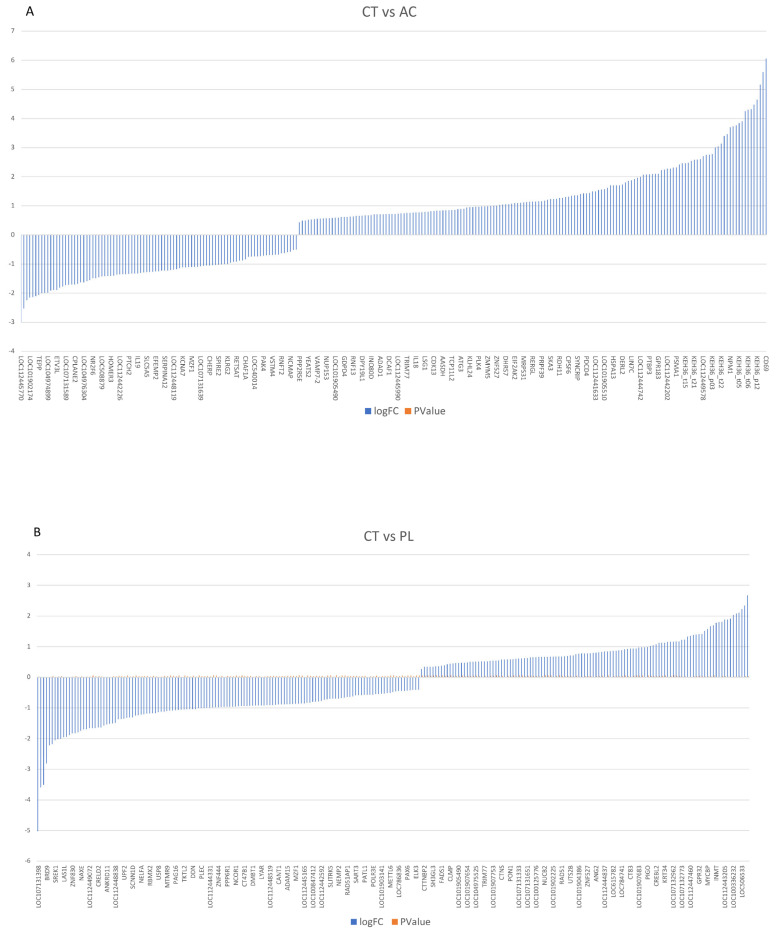

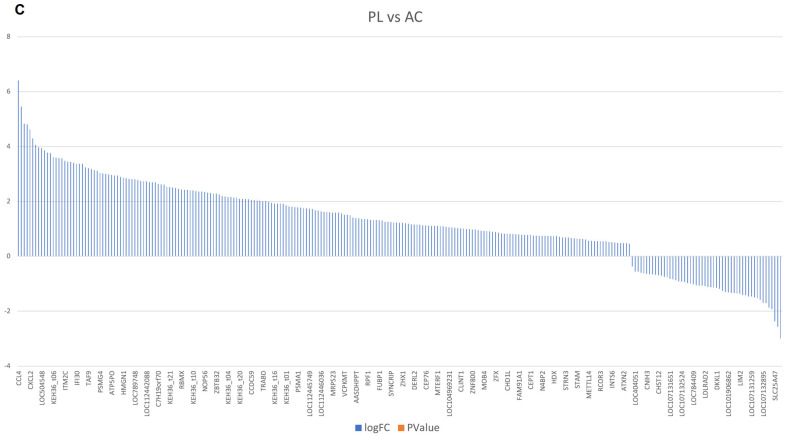
The definition of gene expression analysis among three groups. (**A**) Demonstrate that multiple genes are upregulated and downregulated in the asymptomatic carrier vs. the BLV-negative healthy control group. (**B**,**C**) Compare the hyperactivated and downregulated genes in persistent lymphocytosis and acute-phase proteins, the BLV-negative healthy group, persistent lymphocytosis, and asymptomatic carriers, respectively. CT: BLV-negative healthy group. AC: asymptomatic carrier. PL: persistent lymphocytosis. Log FC: Logarithm fold change, *p* Value: probability value.

Notably, based on our selection criteria, 195 genes were exclusively active in asymptomatic infected samples, while 541 genes were uniquely expressed in non-infected samples. A visual representation of the data (Figure 1A) highlights the differences between infected and non-infected cells. These DEG results are exceptionally differentiated when comparing non-infected cows to those with high positive viral load levels. A total of 22,797 reads were detected, with 9900 genes showing upregulation and 12,897 exhibiting downregulation in PL versus CT ones (Supplementary File S1).

In contrast, 104 genes were exclusively active in persistent lymphocytosis, while 90 genes were uniquely expressed in non-infected samples (Figure 1B,C). Interestingly, a gene expression difference was observed between the AC and PL groups, with 11,696 genes showing overexpression and 11,101 genes exhibiting lower expression in PL compared to asymptomatic carriers. Among them, 758 genes were exclusively active in persistent lymphocytosis, while 109 genes were uniquely expressed in AC samples (Figure 1C).

**Figure 2:**
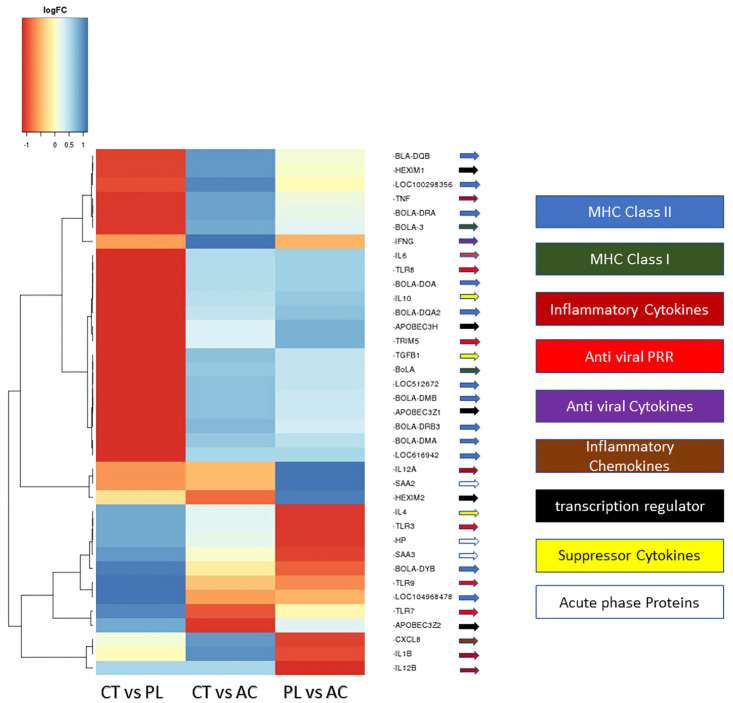
The directional gene expression heatmap analysis of the three study groups. These results demonstrated that gene expression clusters varied in the study groups. CT: BLV-negative healthy group; PL: persistence lymphocytosis; AC: asymptomatic carrier. PRR: pattern recognition receptor. MHC: major histocompatibility complex. Log FC: Logarithm fold change.

In addition, a heatmap clustering analysis was performed to compare the DEG results across all study groups, providing a better understanding of the definitional gene expression block among the involved cases (Figure 2). This map indicates that in persistent lymphocytosis, patients’ *MHC Class II* genes, such as *bovine leukocyte antigen* (*BOLA-DQB*), *BOLA-DRA1*, APOBEC3H*A* and *A2*, *BOLA-DM A* and *B*, *BOLA-DRB3*, and *BOLA-DYB*, as well as transcriptional regulators, such as *HEXIM P-TEFb complex subunit 1* and *2* (*HEXIM1* and *2*), *Apolipoprotein B mRNA Editing Enzyme Catalytic Subunit 3H* (APOBEC3H), and *apolipoprotein B mRNA editing enzyme catalytic subunit 3 Z 2* (APOBEC3Z2), are the most upregulated. Anti-inflammatory cytokines, including Interleukin-4 (IL-4, 10) and Tumor Growth Factor-beta (*TGF-β*), are also upregulated. In contrast, inflammatory cytokines, such as IL-1β and IL-12α, β, antiviral pathogen receptors, such as Toll-Like Receptor (TLR3, 7, and 9), and acute-phase proteins, such as haptoglobin and serum amyloid A2 (SSA2) and A3, are the most downregulated. In addition, Interferon-gamma (IFN-γ), as the main antiviral cytokine, does not exhibit a significant increase. Furthermore, TLR7 and transcription factors, such as APOBEC3Z2 and HEXIM2, are most upregulated in the asymptomatic carrier group compared to the negative control group. At the same time, there is only a partial increase in IL-12α and SSA2, the inflammatory cytokine and the acute-phase protein observed in this group. Moreover, in comparing PL and AC-infected subjects, antiviral receptors, such as TLR3 and 9, acute-phase proteins, such as SSA3 and haptoglobin, and inflammatory cytokines, such as IL-1β and IL-12β, are downregulated in persistent lymphocytosis members (Figure 2).

### 3.3. Functional Analysis

**Figure 3:**
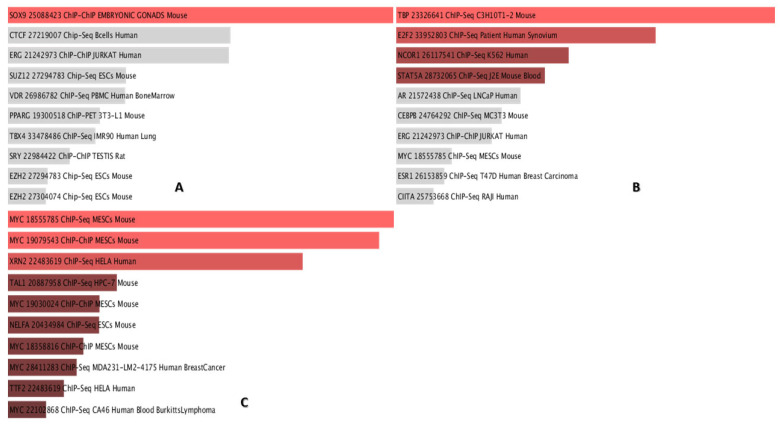
The transcription analysis for the most significant factors that differ between study groups. (**A**) Demonstrated that SOX9 is the only significant transcription factor upregulated in the AC vs. CT group. (**B**) TBP: TATA-binding protein; E2F2: E2F transcription factor 2; NCOR1: nuclear receptor corepressor 1; and STAT5A: signal transducer and activator of transcription 5A. have the highest *p*-value when comparing CT vs. PL individuals. (**C**) The transcription factor analysis demonstrated that MYC: myelocytomatosis oncogene, XRN2, and TAL1 are the most upregulated TFs compared to PL vs. AC. SOX9: SRY-Box transcription factor 9; AC: asymptomatic carrier; CT: control group; PL: persistence lymphocytosis; TFs: transcription factors; XRN2: 5′-3′ exoribonuclease 2; TAL1: TAL BHLH transcription factor 1.

An analysis of gene ontology enrichment revealed that a significant upregulation in the PBLs of asymptomatic carriers is explicitly associated with the SRY-Box transcription factor 9 (SOX9) transcription factor, which plays a role in B-cell development, particularly in terminal differentiation to plasma cells [19,20] (Figure 3A). In comparing the negative control group and persistent lymphocytosis, the TATA-box binding protein, E2F transcription factor 2, nuclear receptor corepressor 1, and signal transducer and activator of transcription 5A are the most upregulated transcription factors (Figure 3B). Furthermore, a CHeA transcription analysis identified that MYC, 5′-3′ exoribonuclease 2 (XRN2), or a Dhm1-like protein and TAL BHLH transcription factor 1 (TAL 1) are upregulated in PL compared to asymptomatic carriers (Figure 3C).

### 3.4. The PPINs Analysis

**Figure 4:**
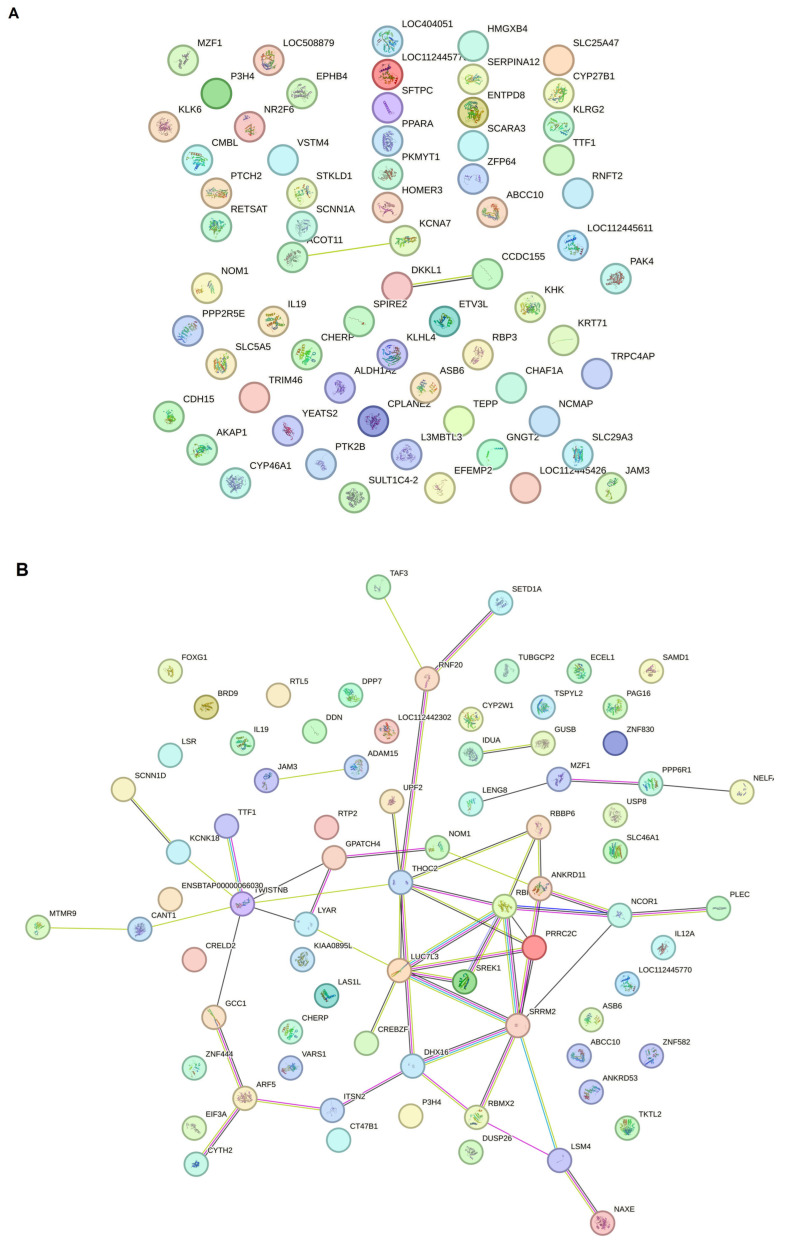

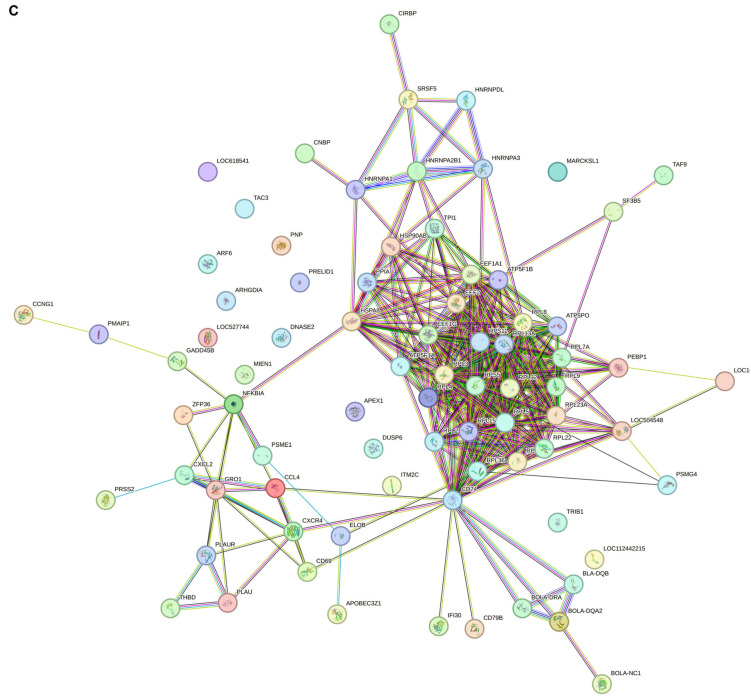
The string network analysis demonstrated that the protein interaction has at least two dense PL cores compared to CT and AC. (**A**) The STRING network analysis between control negative and asymptomatic carriers demonstrated that the protein network does not progress to make new connection pathways despite the significant DEG difference between the two groups. (**B**) The string PPIN analysis compared to persistent lymphocytosis and control negative. According to the increase in DEG variety, the protein network, which may be involved in oncogenesis, is also in progress. (**C**) The PPIN analysis between PL and AC demonstrated that a proportion of proteins start to make a network and progress to the malignant stage compared to the latency period.

In our study, PPINs in three groups were examined and compared. The upregulated genes from each group’s compression were obtained from the DEA file and inserted into the STRING program. For gene selection, a Log FC ≥ 2 was considered the cutoff point; therefore, approximately the top 250 genes are available for creating the network (Figure 4A–C).

### 3.5. Hub Gene Identification

For the clustering gene profile analysis among study groups, Gephi was used to investigate the top-selected genes and understand the critical network (Supplementary File S2).

**Figure 5:**
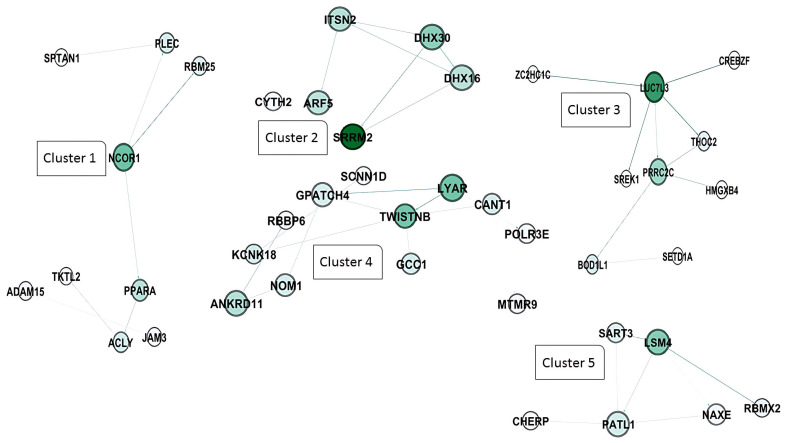
Protein–protein interaction network and cluster analysis of differentially expressed genes. This figure represents a protein–protein interaction (PPI) network constructed from differentially expressed genes grouped into five distinct clusters based on their functional associations. Each node represents a protein, and edges denote predicted or experimentally validated interactions. The intensity of the green color in the nodes corresponds to the degree of connectivity, with darker shades indicating higher interaction scores. Cluster 1 includes NCOR1 (nuclear receptor corepressor 1), PPARA (peroxisome proliferator-activated receptor alpha), PLEC (plectin), RBM25 (RNA binding motif protein 25), SRTAN1 (sirtuin and ankyrin repeat domain containing 1), ACLY (ATP citrate lyase), JAM3 (junctional adhesion molecule 3), TKTL2 (transketolase-like 2), and ADAM15 (ADAM metallopeptidase domain 15). Cluster 2 consists of SRRM2 (serine and arginine repetitive matrix 2), ARF5 (ADP ribosylation factor 5), ITSN2 (intersectin 2), DHX16 (DEAH-box helicase 16), DHX30 (DEAH-box helicase 30), and CYTH2 (cytohesin 2). Cluster 3 includes LUC7L3 (LUC7 like 3 pre-mRNA splicing factor), PRRC2C (Proline-rich coiled-coil 2C), THOC2 (THO complex subunit 2), CREBZF (CREB/ATF bZIP transcription factor), SREK1 (serine/arginine-rich splicing factor 1), ZCCHC14 (zinc finger CCHC-type containing 14), HMGXB4 (high mobility group box family member B4), BOD1L1 (biorientation of chromosomes in cell division 1-like 1), and SETD1A (SET domain containing 1A). Cluster 4 contains LYAR (ly1 antibody reactive), TWISTNB (TWIST neighbor), NOM1 (nucleolar protein with MIF4G domain 1), ANKRD11 (ankyrin repeat domain 11), KCNK18 (potassium two pore domain channel subfamily K member 18), GPATCH4 (G patch domain containing 4), RBBP6 (retinoblastoma binding protein 6), SCNN1D (sodium channel epithelial 1 subunit delta), GCC1 (GRIP and coiled-coil domain containing 1), CANT1 (calcium activated nucleotidase 1), and POLR3E (RNA polymerase III subunit E). Finally, cluster 5 includes SAMD4A (SM4) (sterile alpha motif domain containing 4A), SART3 (squamous cell carcinoma antigen recognized by T cells 3), PATL1 (protein associated with topoisomerase II homolog 1), NAXE (NAD(P)HX epimerase), RBMX2 (RNA binding motif protein X-linked 2), and CHERP (Calcium homeostasis endoplasmic reticulum protein).

For the CT vs. PL study groups, the protein–protein interaction network analysis by Gephi demonstrated that five gene hubs persist in this analysis. The main hubs are nuclear receptor corepressor 1 (NCOR1), serine/arginine repetitive matrix 2 (SRRM2), LUC7 Like 3 pre-mRNA splicing factor (LUC7L3), TWIST NEIGHBOR (TWISTNB), and the LSM4 homolog U6 small nuclear RNA and mRNA degradation associated (LSM4) (Figure 5).

**Figure 6:**
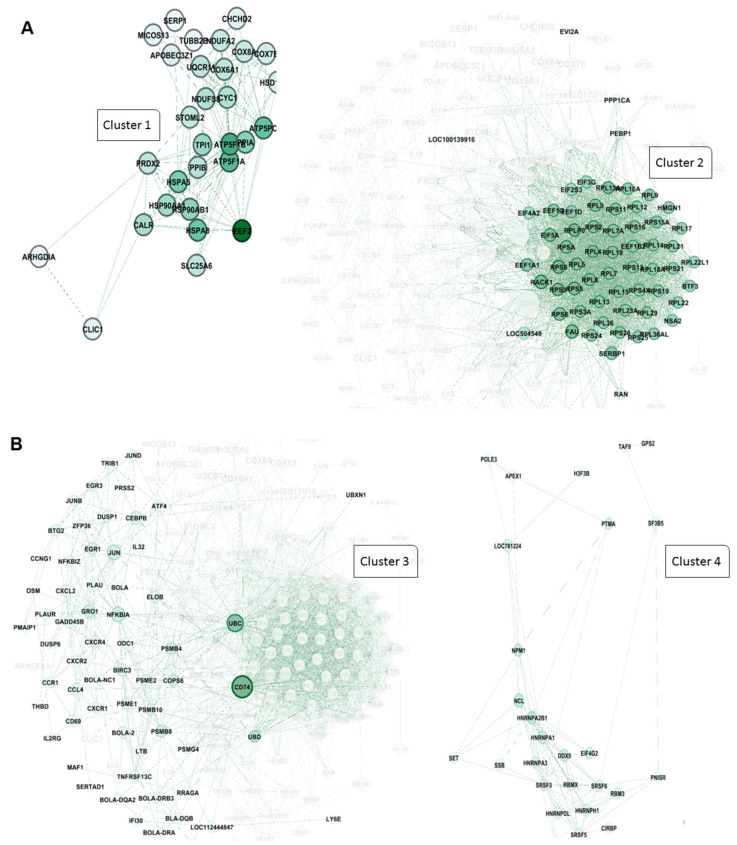
Protein–protein interaction network and cluster analysis of differentially expressed genes. A and 6 show the protein–protein interaction (PPI) networks for two major clusters of genes based on functional grouping and connectivity. Each node represents a protein, while edges denote predicted or experimentally validated interactions. The intensity of the green color of the nodes indicates the degree of connectivity, with darker green nodes representing highly connected hub proteins. (**A**) cluster 1: This cluster primarily consists of proteins associated with mitochondrial function, protein folding, and energy metabolism. Key members include EEF2 (eukaryotic translation elongation factor 2), ATP5F1A and ATP5PB (ATP synthase F1 subunit alpha and ATP synthase peripheral stalk-membrane subunit), PPIB (peptidyl-prolyl cis-trans isomerase B), HSPA5, HSP90AB1, and HSP90AA1 (heat shock proteins involved in protein folding and stress response), CALR (calreticulin), PRDX2 (peroxiredoxin 2), STOML2 (stomatin-like protein 2), TPI1 (triosephosphate isomerase 1), and SLC25A6 (ADP/ATP translocase). Other components include COX6A1, COX8A, and COX7C (cytochrome c oxidase subunits), NDUFS6 (NADH:ubiquinone oxidoreductase subunit), UQCR10 (ubiquinol-cytochrome c reductase), and mitochondrial-associated factors like MICOS13 (MICOS complex subunit). These proteins form a highly connected sub-network involved in oxidative phosphorylation and protein homeostasis. (**B**) cluster 2: This cluster is highly enriched for ribosomal proteins and components of the translational machinery, highlighting its association with protein synthesis and ribosome biogenesis. Key proteins include RPS (ribosomal protein S family: RPS3, RPS4, RPS5, etc.), RPL (ribosomal protein L family: RPL7, RPL10, RPL11, etc.), EIF3G, EIF3F, EIF4A2, and EIF5A (eukaryotic translation initiation factors), and other translation-related proteins, such as EEF1A1 (eukaryotic translation elongation factor 1-alpha). Additional members include RAN (ras-related nuclear protein), FAU (ribosomal protein S30 fusion protein), PEBP1 (phosphatidylethanolamine binding protein 1), and PPP1CA (protein phosphatase 1 catalytic subunit alpha). The dense connectivity of these nodes indicates a critical role in ribosome structure and translational control.

In addition, the clustering analysis revealed that four central hub clusters of proteins facilitated the progression of oncogenesis and cell proliferation in PL vs. AC. These genes are *eukaryotic elongation factor 2* (*EEF2*), *ubiquitin C* (*UBC*), *CD74*, *and heterogeneous nuclear ribonucleoprotein A2/B1* (*HNRNP A2B1*), which are clustered in groups of one to four. The interesting aspect of cluster 2 is that almost all of the genes exhibit the same betweenness, closeness, and degree in the network clustering analysis (Figure 6A,B).

## 4. Discussion

This study investigated the transcriptional landscape of BLV infection in cattle, focusing on the differences between AC, PL, and a BLV-negative healthy group (CT). Our findings reveal significant alterations in gene expression profiles associated with BLV infection severity, highlighting potential mechanisms of viral persistence and disease progression (Table 1).

A substantial number of differentially expressed genes was identified in comparisons across all groups. This difference was even more pronounced when comparing CT to PL animals. These data, visually represented in Figure 1, clearly demonstrate a progression of transcriptional changes correlating with disease severity. Dong. W et al., in their review, declared that viruses manipulate host cells by using their proteins to interfere with the cell’s signaling pathways. This interaction activates downstream signaling cascades, leading to the recruitment of transcriptional machinery to specific genes, ultimately enhancing their expression [15].

The heatmap analysis (Figure 2) provided a comprehensive overview of DEGs’ patterns across groups, revealing key pathways affected by BLV infection. In PL animals, we observed upregulation of *MHC Class II* genes, transcriptional regulators, such as *HEXIM1/2* and *APOBEC3* (involved in antiviral defense and RNA editing), and anti-inflammatory cytokines (*IL-4*, *IL-10*, *TGF-β*). Conversely, we found downregulation of pro-inflammatory cytokines (*IL-1β*, *IL-12α/β*), antiviral pathogen receptors (*TLR3*, *7*, *9*), and acute-phase proteins (haptoglobin, SAA2/3). Based on these results, it is understandable that BLV in the lymphocytosis stage changes the immune responses from cellular immunity to humoral immunity by hijacking the MHC class II molecules, decreasing cellular immunity cytokines, and increasing humoral cytokines, such as IL-4 and IL-10 [21]. Also, it activated the B cell more than the T-cell, which helped the BLV increase its number of infected cells. Additionally, an increase in TGF-β shifts the immune response from an inflammatory reaction to an anti-inflammatory response, which helps the virus survive.

In contrast, when using the BLV vaccine peptide, the best result is an induced Th-1 response, accompanied by a decrease in IL-4 and IL-10 [22]. Despite the APOBEC functions in deaminating viral RNA, generating C to U changes, and inhibiting proviral integration and reverse transcription, a previous study demonstrated that an increase in this gene expression is non-correlated with its known antiviral function in BLV infection [18]. Takafumi Shichijo et al. (2024) stated that *Deltaretroviruses*, such as HTLV, can utilize APOBEC molecules to enhance their proliferation and pathogenicity of infection [23]. Interestingly, their reported increase in TGF-β expression would help the HTLV utilize this protein for viral proliferation [23].

In addition, Shichijo T. et al. in 2024 investigated the role of the host enzyme APOBEC3G, a cytidine deaminase involved in antiviral defense, in shaping the pathogenicity of deltaretroviruses, such as HTLV and BLV [23]. The researchers demonstrate that deltaretroviruses exhibit varying susceptibility to APOBEC3G-mediated mutagenesis, which can influence viral replication and persistence. Viruses that efficiently evade APOBEC3G activity are more likely to establish chronic infections and contribute to oncogenesis [23]. This study highlights the interplay between viral factors and host restriction mechanisms, suggesting that APOBEC3G vulnerability is a critical determinant of deltaretroviral pathogenic potential and a possible target for therapeutic interventions.

The relatively modest increase in IFN-γ, a crucial antiviral cytokine, suggests a potential immune evasion strategy employed by BLV. Interestingly, the AC group showed upregulation of E2F2, APOBEC3Z2, and HEXIM2, suggesting a distinct early immune response compared to PL. The differences in the expression of inflammatory cytokines and acute-phase proteins between the PL and AC groups further support this notion [18]. Compared to AC, downregulation of TLR3, TLR9, SAA3, haptoglobin, IL-1β, and IL-12β in PL points to possible immune exhaustion or suppression characteristic of advanced disease.

Gene ontology enrichment analysis (Figure 3) further illuminated the functional implications of these transcriptional changes. In AC, the significant upregulation of SOX9, a key regulator of B cell differentiation, suggests potential alterations in B cell development and function [24,25]. SOXs can affect the establishment of viral latency, where the retroviral genome is integrated into the host genome but not actively producing viral proteins. By influencing the expression of host genes involved in DNA repair and epigenetic modification, SOXs can help create an environment that favors the persistence of the viral genome in a latent state during BLV infection among asymptomatic carriers [26].

In contrast, the comparison between CT and PL highlighted the upregulation of transcription factors (TATA box binding protein, E2F2, NCOR1, STAT5A), potentially reflecting the dysregulation of cellular processes. Both STAT5A and E2F2 regulate genes involved in cell growth and differentiation. In addition, the TATA box binding protein can activate the expression of multiple genes. Finally, NCOR1 can be activated by histone deacetylases (HDACs), leading to chromatin condensation and transcriptional repression [27,28,29,30]. In 2007, Saenz-Robles et al. reported that the superantigen of Simian virus 40 could activate E2F2, inducing T-cell proliferation in infected cells and potentially leading to neoplastic transformation [31].

Furthermore, the increased expression of STAT5A and STAT5B was previously reported in a study by Yamada K. et al., which evaluated this gene expression in leukemia cell lines [32]. The CHeA analysis revealed the upregulation of MYC, XRN2, and TAL1 in PL compared to AC, suggesting that these genes are involved in the progression to persistent lymphocytosis. Stone D. M. et al. state in their study that c-myc, as a proto-oncogene alongside pim-1, is upregulated in BLV persistent lymphocytosis animals and can induce a preneoplastic B cell condition in infected subjects [33]. Despite the protective role of 5’ exoribonucleases Xrn2 in RNA viral infections, a recent study demonstrated its upregulation in respiratory syncytial virus and Hepatitis C virus, which may suggest a dual function in an increased number of viruses [34,35].

Therefore, our study provides a detailed transcriptomic analysis of BLV infection, revealing distinct gene expression signatures associated with different stages of the disease. The observed alterations in immune response, antiviral defense, and cellular regulation highlight potential mechanisms underlying BLV pathogenesis and may offer novel therapeutic targets for this significant bovine pathogen.

This study further investigated the PPINs to understand the relationships between DEGs and disease progression in BLV infection. The comparison between CT and AC (Figure 4A) revealed a significant difference in DEGs. However, the resulting PPIN did not exhibit extensive new connections or pathways, suggesting a limited level of interaction amongst the upregulated genes at this early stage of infection. In contrast, the PPIN analysis comparing CT and PL groups (Figure 4B) demonstrated a more complex network with increased connections, suggesting more extensive protein interactions and a possible link to oncogenesis. This expansion of the interaction network was further amplified in the comparison between PL and AC groups (Figure 4C), revealing a substantial increase in protein interactions and the development of a network indicative of progression to a malignant stage, contrasting sharply with the latency period represented by the AC group. Our results demonstrated that the malignant stage occurs when PPINs form new connections to activate steady-state cells and transform them into cells with abnormal proliferation in BLV infection.

The clustering analysis (Figure 5) demonstrated that five genes emerged as significant hubs: NCOR1, SRM2, LUC7U3, TWIST1, and LSM4. These hubs are likely central to the extensive network changes observed at this advanced stage of the disease. We previously mentioned the role of NCOR1 in controlling gene expression as an epigenetically active element. SRRM2 is one of the foremost components in pre-mRNA splicing and nuclear speckle formation, which is expressed on the surface of most cancerous cells [36]. Usama Ashraf et al. in 2018 stated that SRRM2 can carry out host splicing variation in HIV-infected subjects to reduce the innate immune response and increase cellular damage [37]. In addition to the 5′ pre-mRNA splicing factor, the 3′ end splicing elements, such as LUC7U3, showed hub interactivity in the PL group compared to HC members, which is reported to increase in other types of cancer, such as myeloid neoplasms and ovarian cancer [38,39]. In addition, a 3′-terminal oligo(U) tract of U6 snRNA, LSM4, which plays a role in pre-mRNA splicing by activating U4/U6 snRNP formation [40], is also involved in BLV pathogenesis in the PL group. Chen L. et al. and Yin J. et al., in their studies on hepatocellular carcinoma and breast cancer, respectively, highlighted the role of LSM4 as a hub gene candidate or one that is overexpressed in their patients [41,42]. RNA metabolism and maturation are among the most activated pathways during the lymphocytosis stage of BLV infection. Besides the RNA splicing and maturation process, the DNA-dependent RNA polymerase element TWISTNB, which catalyzes the transcription of DNA into RNA, is considered the primary mediator in the proliferation of the viral genome. It is also considered a potential upregulated gene in head and neck squamous cell carcinomas and breast cancer [43,44].

Furthermore, the PL vs. AC comparison (Figure 6) revealed four major protein clusters strongly implicated in oncogenesis and cell proliferation. These clusters centered on the hub genes EEF2, UBC, CD74, and HNRNPA2B1. NRNPA2B1 can play a pivotal role in RNA processing, splicing, and translation, which increases the expression of this protein observed in another type of lymphoma [45]. Moreover, Zuo D. et al. (2023) declared that the use of an HNRNPA2B1 agonist effectively inhibits HBV and SARS-CoV-2 Omicron infection [46]. Therefore, this protein can play an essential role in viral replication. Besides the RNA metabolism and maturation process in BLV pathogenesis, protein expression regulation is another pathway involved. EEF2 is the primary step of the ribosomal movement from the A site to the P site, whose increased expression leads to increased protein production in the cell. At the same time, Valiente-Echeverria F. et al. reported that HIV-1 gag blocks stress granule assembly irrespective of eIF2α in response to viral infection and facilitates HIV virion production [47]. The UBC gene, also known as Ubiquitin C, is one of four genes in the human genome that encode the protein ubiquitin. Ubiquitin is crucial in various cellular processes, including protein degradation, DNA repair, and cell cycle regulation [48,49]. In addition, we found that another element involved in MHC Class II antigen presentation, CD74, can play a role as a hub gene in PL individuals. Previous studies have stated that HIV-derived proteins, such as Vpu and glycoprotein 41, could interact with CD74 and increase the viral load [50,51].

Intriguingly, cluster 2 showed remarkably similar betweenness, closeness, and degree centrality scores for its constituent genes, highlighting a high degree of interconnectedness and functional coherence within this cluster.

## 5. Conclusions

This study provides an integrated transcriptomic and network-based analysis of BLV infection, revealing major transcriptional reprogramming from asymptomatic carriers to persistent lymphocytosis. We identified key hub genes (*NCOR1*, *SRRM2*, *LUC7L3*, *TWISTNB*, *LSM4*, *EEF2*, *UBC*, *CD74*, and *HNRNPA2B1*) associated with oncogenesis and immune modulation, highlighting their potential roles in BLV pathogenesis. These findings support the use of combined transcriptomic and protein interaction analyses to uncover critical molecular drivers of disease. Future research should focus on validating the roles of these hub genes in BLV infection and exploring their potential as biomarkers for disease prognosis and treatment monitoring. Larger sample sizes and the integration of additional omics data, such as proteomics and metabolomics, will further refine our understanding of BLV–host interactions and pave the way for the development of effective control strategies.

## Figures and Tables

**Table 1:** Summary of key gene/pathway and hub gene changes in BLV infection.

Comparison	Upregulated Genes/Pathways	Downregulated Genes/Pathways	Hub Genes (PPIN Analysis)	Functional Category	Notes
CT vs. AC	TLR7, APOBEC3Z2, HEXIM2	Partial decrease in IL-12α, SSA2	No major hubs identified	Innate immune activation, antiviral defense	Suggests early immune activation in asymptomatic carriers
CT vs. PL	*MHC Class II* (BOLA genes), HEXIM1/2, APOBEC3H/Z2, IL-4, IL-10, TGF-β	IL-1β, IL-12α/β, TLR3,7,9, haptoglobin, SAA2/3	NCOR1, SRRM2, LUC7L3, TWISTNB, LSM4	Adaptive immunity shift, anti-inflammatory signaling, RNA processing	Indicates immune suppression and humoral bias in PL stage
PL vs. AC	Distinct activation of protein metabolism genes	TLR3,9, haptoglobin, SAA3, IL-1β, IL-12β	EEF2, UBC, CD74, HNRNPA2B1	Oncogenic progression, protein translation, RNA processing	Represents transition toward malignancy-like changes

AC: asymptomatic carrier; CT: control group; PL: persistence lymphocytosis; HEXIM1/2: hexamethylene bis-acetamide inducible protein 1; APOBEC3H/Z2: apolipoprotein B mRNA editing enzyme catalytic subunit 3H/Z2; BOLA: butyrophilin subfamily 1 member A; IL: interleukin; TGF-β: tumor growth factor beta; TLR: Toll-like receptors; SSA: serum amyloid A2; NCOR1: nuclear receptor corepressor 1; SRRM2: serine and arginine repetitive matrix 2; LUC7L3: LUC7 like 3 pre-mRNA splicing factor; TWISTNB: TWIST neighbor; LSM4: U6 snRNA-associated Sm-like protein; EEF2: eukaryotic elongation factor 2; UBC: ubiquitin C; CD74: cluster of differentiation 74; HNRNPA2B1: heterogeneous nuclear ribonucleoprotein A2/B1.

## Data Availability

The datasets used and analyzed during the current study are available from the corresponding author upon reasonable request.

## Supplementary Materials

The following supporting information can be downloaded at https://www.mdpi.com/article/10.3390/pathogens14090887/s1, Supplementary File S1 contains three Excel files comparing gene expression in healthy control (CT) versus asymptomatic carrier (AC), healthy control versus persistence lymphocytosis (PL), and persistence lymphocytosis versus asymptomatic carrier. The files contain information about the gene symbol, logFC, log CPM, F, *p*-value, for each group. In addition, The Excel files also include the 250 gen with highest logFC and lowest *p*-value. In addition, the Supplementary File S2 contained the cluster analysis of CT versus AC, CT versus PL, and PL versus AC to detect the hub genes and provide the protein-protein interaction network values. Each file includes: Id, Label, indegree, outdegree, degree, weighted indegree, weighted outdegree, Weighted degree, Eccentricity, closnesscentrality, harmonicclosnesscentrality, betweenesscentrality, modularity_class, eigencentrality, stat_inf_class, X, Y, Size, and Color.
